# SHARE – workpackage 5: evidence based recommendations for diagnosis and treatment of juvenile localized scleroderma and juvenile systemic sclerosis

**DOI:** 10.1186/1546-0096-12-S1-P117

**Published:** 2014-09-17

**Authors:** Roberta Culpo, Sebastian J Vastert, Angelo Ravelli, Nico Wulffraat, Ivan Foeldvari, Francesco Zulian, Jordi Anton, Tadej Avcin, Eileen Baildam, Christina Boros, Jeffrey Chaitow, Tamas Constantin, Pavla Dolezalova, Ozgur Kasapcopur, Sheila Knupp de Oliveira, Clarissa Pilkington, Annett van Royen, Ricardo Russo, Claudia Saad-Magalhaes, Natasa Toplak

**Affiliations:** 1 Paediatric Rheumatology Unit, University of Padua, Padua, Italy; 2 Department of Pediatric Immunology and Rheumatology, Wilhelmina Children's Hospital, Utrecht, Netherlands; 3 Paediatric Rheumatology Unit, Istituto Gaslini, University of Genoa, Genoa, Italy; 4 Pediatric Rheumatology Unit, Hamburg, Germany

## Introduction

Juvenile Localized Scleroderma (JLS) and Juvenile Systemic Sclerosis (JSSc) form a group of rare pediatric diseases that can lead to significant morbidity. Evidence-based guidelines are sparse and management is mostly based on physician’s experience. Consequently, treatment regimens differ throughout Europe. In 2012, a European initiative called SHARE (Single Hub and Access point for pediatric Rheumatology in Europe) was launched to optimize and disseminate diagnostic and management regimens in Europe for children and young adults with rheumatic diseases.

## Objectives

One of the aims of SHARE was to provide evidence based recommendations for diagnosis and treatment of Juvenile Scleroderma.

## Methods

Evidence based recommendations were developed using the European League Against Rheumatism (EULAR) standard operating procedure. An expert committee was instituted, consisting of pediatric rheumatologists and experts in Juvenile Scleroderma. The expert committee defined search terms for the systematic literature review. Two independent experts scored articles for validity and level of evidence. Recommendations derived from the literature were evaluated by an online survey. Those with less than 80% agreement during the online survey were reformulated. Subsequently, all recommendations were discussed at a consensus meeting using the nominal group technique [Delbeq AL. A group process model for problem identification and program planning *The Journal of Applied Behavioral Science July 1971 7: 466-492*]. Recommendations were accepted if more than 80% agreement was reached.

## Results

The literature search yielded 1550 articles for JLS and 8562 for JSSc, of which 52 and 37, respectively (25 for diagnosis and 27 for treatment of JLS, 21 for diagnosis and 16 for treatment of JSSc) were considered relevant and therefore scored for validity and level of evidence. 42 articles (15 for diagnosis and 14 for treatment of JLS, 6 for diagnosis and 7 for treatment of JSSc) were scored valid and used in the formulation of the recommendations. 11 recommendations for diagnosis and 7 for treatment were suggested in the online survey on JLS. Ten recommendations for diagnosis and 6 for treatment were accepted with more than 80% agreement after the consensus meeting. Six recommendations for diagnosis and 5 for treatment were suggested in the online survey on JSSc. 6 recommendations for diagnosis and 4 for treatment were accepted with more than 80% agreement after the consensus meeting. Topics covered for diagnosis and for treatment were, for JLS, disease activity assessment and response to therapy, disease severity and damage assessment, extra-cutaneous involvement (articular, musculoskeletal, neurological, ophtalmological, dental, maxillo-facial), topical treatments (medium-dose UVA1 phototherapy, imiquimod) and systemic treatment (corticosteroids, metotrexate, mychophenolate mofetil); for JSSc disease severity and damage assessment, diagnosis of skin involvement, lung involvement and Raynaud's phenomenon, treatment of skin, lung and vascular involvement and autologous stem-cell transplantation.

## Conclusion

The SHARE initiative provides recommendations for diagnosis and treatment for Juvenile Scleroderma and thereby facilitates improvement and uniformity of care throughout Europe.

## Disclosure of interest

None declared.

